# Increased research productivity in Nigeria: more to be done

**DOI:** 10.4155/fsoa-2018-0083

**Published:** 2019-01-25

**Authors:** Olumide Adedokun Odeyemi, Olubukola Adeniyi Odeyemi, Florence A Bamidele, Otunba Ahmed Adebisi

**Affiliations:** 1 Ecology & Biodiversity Centre, Institute for Marine & Antarctic Studies (IMAS), University of Tasmania, Launceston, Australia; 2 Food Safety & Quality Unit, Centre for Research, Training & Development, Higis International Foundation, Nigeria; 3 Public Health Unit, Centre for Research, Training & Development, Higis International Foundation, Nigeria; 4 Department of Biological sciences, School of Applied Science, Yaba College of Technology, Lagos, Nigeria; 5 Daniel & Fola Biotechnology Foundation, Lagos, Nigeria

**Keywords:** collaboration, Nigeria, research and development, research output, research productivity

Research and experimental development is the explorative, innovative and systematic creation of new, or the extension of existing, knowledge to improve humanity, culture and society [1–3]. Research outputs that are either traditional or nontraditional are used as major yardsticks in measuring the scientific and technological advancement of any nation. Traditional research outputs include the generation or improvement of processes, products, policies, concepts, patents, publications and books; while nontraditional research outputs include visual art work, design/architectural work, textual work, film/video, performance, web-based exhibition and so on. The focus of this article is on traditional research outputs [2,4].

Research is about solving a problem, obtaining new facts or adding information to elucidate a problem. It is about inquisitiveness and the ability to look at problems from different angles. Any country that does not believe in research will remain underdeveloped. In Nigeria, research and higher institutions of learning have been poorly funded. Laboratories are poorly equipped with most of the equipment lying obsolete, no training for staff and a lack of basic amenities. How do our products and researchers compete with their contemporaries in developed countries? Most of our research is not translational because there is little collaboration between the industries, research institutions and higher institutions.

## Research productivity in Nigeria

Research productivity over the years has been measured by the number of peer-reviewed articles published by academics and/or scientists in institutions such as universities and/or research institutes [5]. A recent report investigated the major trend of scientific research from six selected West African countries demonstrated that there has been increase in scientific publications in Nigeria over the past 16 years [6]. For example, there was an increase in publications, from 2500 articles in 2008 to over 4000 in 2017, comprising more than half the total volume of publications from West Africa. There were more publications from Nigeria than any other West African country. This increase could partly be because Nigeria is the most populous country in Africa and has the highest number of higher education institutions such as universities, polytechnics, monotechnics and research institutes. Most of the research publications reported in Nigeria were from the public, environmental, occupation and health domains [6]. According to the report, beside research from infectious diseases and plant sciences, the least prominent research outputs were from pharmacology, food science and technology domains and when compared with other West African countries such as Ghana, Niger, Burkina Faso, Senegal and the Ivory Coast, Nigeria has the least research impact [6]. Similarly, Nigeria had the fewest international collaboration over this period. The University of Ibadan (Ibadan, Nigeria) was reported to have the highest number of publications and international research collaborations [6]. This implies that collaboration with researchers from other parts of the world helps increase research impact. However, most (over 1000) of the collaborations from Nigeria were with researchers from the USA, UK, South Africa and Malaysia. The reasons behind this observation are easy to glean. The USA is known globally for its quality education and research and has over 10,000 Nigerian students in 2017 [7]. According to US Embassy in Nigeria, the USA awarded $9 million USD in scholarship and financial aid to Nigerian students in 2017, enabling them to study in the USA.

## Factors influencing research productivity in Nigeria

Despite the increase in research productivity evidenced by publications in Nigeria, how many of the research publications have been translated into policy, commercialized products, patents or processes that have improved, or have the potential to improve, the standard of living through poverty reduction, creation of jobs and prevention of infections and diseases? For example, although there are various publications on food safety in Nigeria, the policy impact of this cannot be felt in society. It is therefore not enough to carry out research and publish the outcome; it is imperative that the outputs translate beyond academic or research exercise. This is because when properly harnessed, research productivity can enhance the quality of teaching and career development that can influence production of graduates that can translate acquired knowledge to socioeconomic development [8]. Hence, there is more to be done both at the federal and institutional level, including setting up a functional national research council that will oversee research impacts and productivity in Nigeria, encouraging research collaboration at national and international levels. Research collaboration includes collaboration between individuals (interindividual), groups, departments (interdepartment), institutions (interinstitutional) and discipline (inter/multi-disciplinary) at the national and international levels [9]. This could be done through encouragement of multidisciplinary research and intellectual exchange based on the Field of Science and Technology classification [10]. Recently, researchers at the Department of Epidemiology, Helmholtz Centre for Infection Research collaborated with Nigerian scientists to develop a mobile application called Surveillance, Outbreak Response Management and Analysis System (SORMAS) that has been used in 15 out of 36 States in Nigeria [11]. SORMAS enables real-time collection and sharing of data on infectious diseases between medical specialists.

Establishment of a national publications database as observed in developed countries is required. For example, Australia and Malaysia both have a national publications database that publication outputs submitted and easily monitored. Universities in Australia usually ensure publications by their researchers are provided to the publication units of the universities which are then forwarded to higher education research data collection (HERDC). In addition, development of robust performance measures like the Research Excellence Framework developed in the UK [12] and the Excellence in Research for Australia [13]. A nationwide study on determinants of research productivity, accessibility to journals, staff, and student exchange programs with universities and research institutes in developed countries, promotion and establishment of multi, trans and interdisciplinary international research collaborations, and partnership with industry through translational and contract research. An agency to set up by the Federal Ministry of Science and Technology in conjunction with nongovernmental organizations could help in achieving all these.

## Conclusion

There has been increase in research activities and attendant publication output in Nigeria. However, this is not equal across disciplines, as more publications are seen in life and health sciences compared with social sciences and engineering. More needs to be done as currently most reported research has not impacted either society or the standard of living for the average Nigerian. As research productivities promote economic growth, it should not be based on numbers of publications only but by impact of such research outputs in ensuring improved quality of life through policy formulations. There is need for adequate funding of research, education, training and infrastructure in Nigerian higher institutions of learning and research institutes. Encouragement of research collaboration and incentives for publishing in legitimate as compared with predatory journals will also motivate translational research outputs.
